# The familial Mediterranean fever (FMF) 50 score: does it work in a controlled clinical trial? Re-analysis of the trial of rilonacept for patients with colchicine resistant or intolerant FMF

**DOI:** 10.1186/1546-0096-13-S1-P158

**Published:** 2015-09-28

**Authors:** P Hashkes, B Huang

**Affiliations:** 1 Shaare Zedek Medical Center, Jerusalem, Israel; 2 Cincinnati Children's Hospital Medical Center, Cincinnati, OH, USA

## Background

The familial Mediterranean fever 50 score (FMF50) was recently devised to define response to treatment and as an outcome measure for clinical trials of FMF.

## Objectives

To examine the performance of the FMF50 score in a previously published trial of rilonacept [1] for patients whose FMF was resistant or intolerant to colchicine.

## Methods

We reanalyzed the data from the controlled trial of rilonacept vs. placebo in 14 patients with colchicine-resistant or intolerant FMF using the FMF50 score as the primary outcome. The FMF50 score required improvement by ≥50% in five of six criteria (attack frequency, attack duration, global patient assessment, global physician assessment, frequency of attacks with arthritis, and levels of acute-phase reactants) without worsening of the sixth criterion.

## Results

In the original trial rilonacept was considered effective according to the primary outcome measure (differences in the attack frequency) with eight analyzable patients considered responders and four as non-responders. According to the FMF50 score, only two participants would have been considered responders to rilonacept, and one to placebo. Only two participants had ≥50% differences between rilonacept and placebo in five criteria. The major explanation for non-response to treatment was that with rilonacept the duration of attack decreased by ≥50% in only 2 participants and 5 participants had no attacks of arthritis either during screening (before randomization) or during treatment with rilonacept.

## Conclusions

The proposed FMF50 score did not differentiate well between responders and non-responders compared to the a priori defined primary outcome measure in this successful controlled study and should be revisited prior to adoption as a primary outcome measure in multinational FMF trials.
